# Construct-to-Construct Internal Distraction and Compression Technique for Scoliosis Correction

**DOI:** 10.3390/jcm14196939

**Published:** 2025-09-30

**Authors:** J. Manuel Sarmiento, Rodrigo Navarro-Ramirez, Hoon Choi, Anthony S. Rinella, Han Jo Kim, Lawrence G. Lenke, Michael G. Vitale

**Affiliations:** 1 Pauline Braathen Neurological Center, Department of Neurosurgery, Cleveland Clinic Florida, 2950 Cleveland Clinic Blvd, Weston, FL 33331, USA; 2 Duly Health and Care, 120 Spalding Drive, Suite 400, Naperville, IL 60540, USA; 3 Hospital for Special Surgery, Spine and Scoliosis Service, 535 East 70th Street, New York, NY 10021, USA; 4 Department of Pediatric Orthopaedics, New York-Presbyterian Morgan Stanley Children’s Hospital, 3959 Broadway-8 North, New York, NY 10032, USA

**Keywords:** construct to construct, internal distraction, internal compression, spinal deformity correction techniques, scoliosis correction

## Abstract

**Background**: Temporary internal distraction is a safe surgical technique that has been shown to improve correction of severe scoliosis. The traditional surgical adjunct for scoliosis treatment in the perioperative period is halo gravity traction, but there are several known disadvantages of this technique. We describe the technical nuances of temporary internal distraction using the construct-to-construct technique, a surgical adjunct that utilizes two rods joined by lateral domino connectors to enact powerful internal distraction or compression forces on the spine for achieving spinal deformity correction. **Methods**: This study was designed as a retrospective review and illustrative surgical technique report. The primary aim was to describe the construct-to-construct internal distraction and compression technique for scoliosis correction, with illustrative models and representative clinical cases. **Results**: Internal distraction using the construct-to-construct configuration is performed early in the surgery to take advantage of the viscoelastic properties of the spine as gradually increasing distraction forces are applied. The surgical technique for applying internal distraction and compression using the construct-to-construct configuration is discussed in detail. **Conclusions**: Construct-to-construct internal distraction and compression techniques are powerful methods to correct severe scoliosis curves, serially distract traditional growing rod constructs, and close three-column osteotomies.

## 1. Introduction

Internal distraction techniques have been shown to be safe and effective for scoliosis correction compared with halo gravity traction (HGT). HGT has traditionally been used as an adjunct perioperative treatment for severe scoliosis curves that exceed 90 degrees [1]. However, HGT is associated with several disadvantages, including prolonged hospital stay, pin site complications, and decreased effectiveness, in correcting lumbar scoliotic curves [2]. Temporary internal distraction techniques have achieved outcomes of 80% mean final curve correction in patients with a mean preoperative curve of 104 degrees [3]. Internal distraction techniques may be performed in a single surgery or in two electively staged operations.

Internal distraction and compression for scoliosis correction can be performed utilizing various techniques. Furthermore, several different anchor points at the proximal and distal ends of the spinal construct can be chosen. For example, proximal anchor points can be pedicle screws, upgoing infralaminar hooks, upgoing transverse process hooks, or upgoing rib hooks [4]. Distal anchor points include lumbar pedicle screws, sacral screws, or iliac wing fixation at the pelvis. This report will focus on describing the construct-to-construct technique that utilizes two rods joined by lateral domino connectors to enact powerful internal distraction or compression forces on the spine for achieving spinal deformity correction.

## 2. Methods

### 2.1. Study Design

This study was designed as a retrospective review and illustrative surgical technique report. The primary aim was to describe the construct-to-construct internal distraction and compression technique for scoliosis correction, with illustrative models and representative clinical cases.

### 2.2. Illustrative Models

To provide clear visualization of the construct-to-construct setup, we used commercially available synthetic sawbone spine models (Sawbones, Vashon, WA, USA). These models were instrumented according to the described technique, and standardized photographs were obtained to create step-by-step illustrative figures.

## 3. Results

### 3.1. Surgical Technique

**Figure 1:**
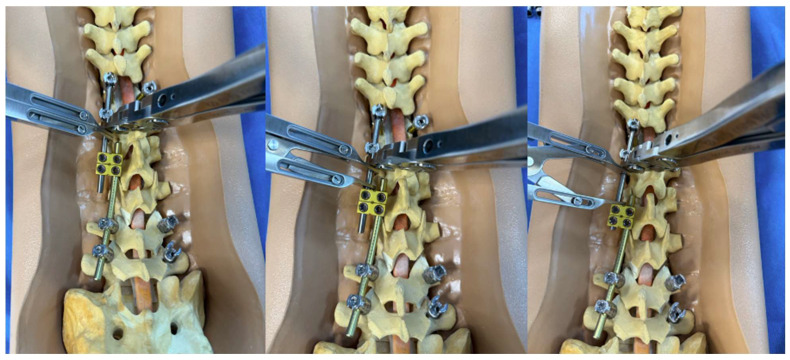
Sawbone model utilizing a construct-to-construct internal distraction technique showing the greater overlap of the rods across the lateral domino connectors, the more “runway” there is to apply distraction.

**Figure 2:**
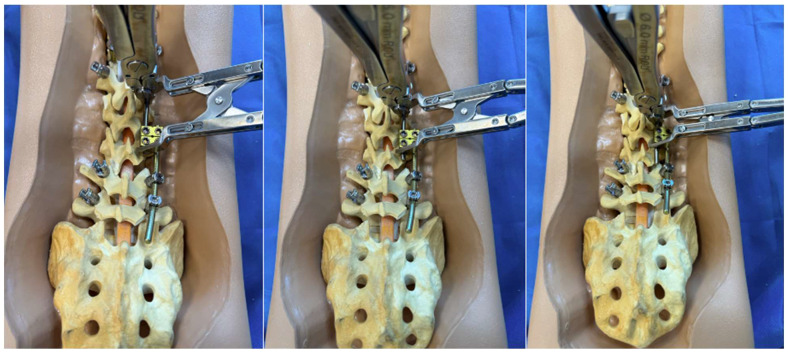
Sawbone model utilizing a construct-to-construct internal compression technique showing reduced overlap of the rods across the lateral domino connectors, the more “runway” there will be to apply compression.

A detailed surgical technique guide by Buchowski et al. in 2007 describes temporary internal distraction in a broader context and highlights important considerations, such as preoperative surgical planning, patient positioning, and placement of proximal and distal anchor points [4]. However, this report aims to focus on the placement and configuration of spinal rods for internal distraction or compression utilizing the construct-to-construct technique. To set up the construct-to-construct configuration, one rod is attached to the proximal anchor points and a second rod to the distal anchor points. There should be at least two (or more) anchor points at the proximal and distal ends to set up this technique. This helps prevent plowing of pedicle screws by distributing the distractive and compressive forces across several motion segments along the spine. These rods are joined by one or two lateral domino connectors to complete the construct-to-construct setup. The more overlap of the rods, the more “runway” there is to apply distraction (Figure 1). Conversely, the less overlap that exists between these two rods, the more “runway” there will be to apply compression (Figure 2). If internal distraction is desired, this construct-to-construct configuration will need to be set up on the concavity of the scoliosis curve. Alternatively, the construct-to-construct configuration will need to be set up on the convexity of the scoliosis curve if internal compression is needed. Distraction or compression is then applied to these rods in a serial, “click-by-click” fashion after loosening the ipsilateral set caps on the domino connectors. Rod grippers may be used to distract or compress against instead of utilizing pedicle screw tulips in order to reduce loosening of the anchor points. After the desired amount of deformity correction is achieved, the set caps on the domino connectors are tightened to hold the correction.

### 3.2. Internal Distraction on the Concavity of a Major Scoliosis Curve

**Figure 3:**
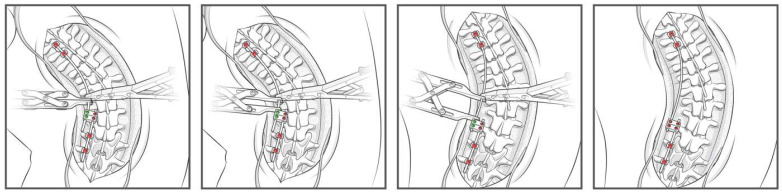
Step-by-step schematic of the construct-to-construct internal distraction technique. The green caps represent loose set caps. Red caps represent tight set caps.

**Figure 4:**
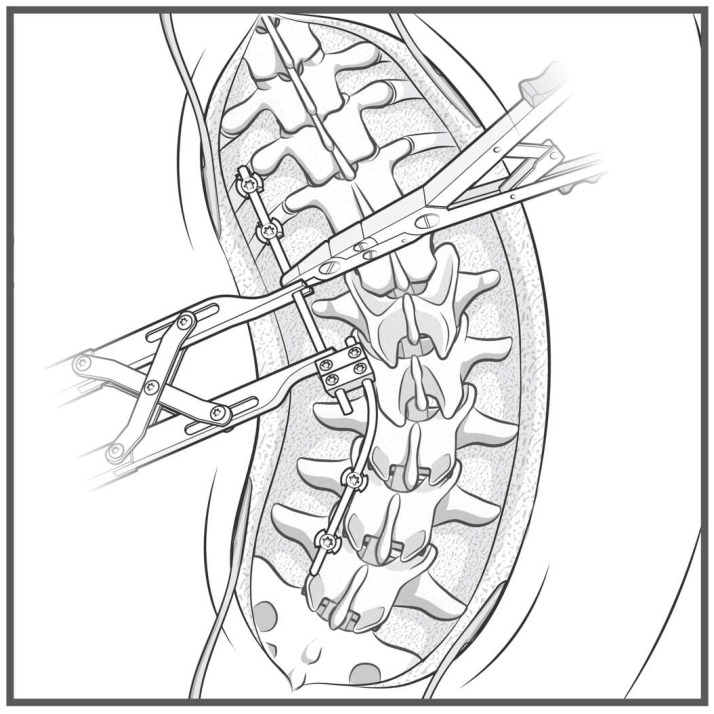
Schematic of the construct-to-construct internal distraction technique with proximal anchor points at thoracic ribs using upgoing rib hooks.

**Figure 5:**
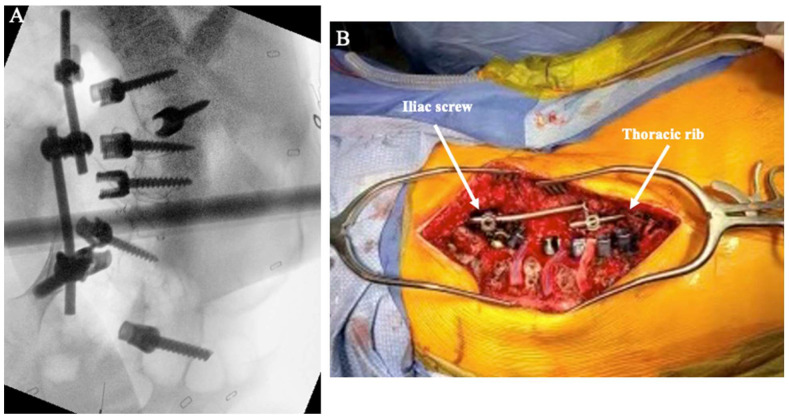
Intraoperative X-ray showing a construct-to-construct internal distraction technique with an iliac screw serving as the distal anchor point and the last thoracic rib serving as the proximal anchor point (**A**). Intraoperative photo showing the iliac screw to thoracic rib setup before distraction is performed (**B**).

The first clinical scenario where the construct-to-construct internal distraction technique may be useful is during surgical correction of severe scoliosis curves > 90 degrees. This technique is most effective in large, flexible sweeping curves that involve multiple spinal levels. Sharply angulated and rigid curves with high-deformity angular ratios (DAR) are better managed with three-column osteotomies [5,6]. We prefer placing our anchor points to set up the construct-to-construct internal distraction configuration early in the procedure. The viscoelastic environment of the spine will permit gradual sequential distraction to relax soft tissues and make them more compliant with progressive correction techniques. In severe major curves, it is often challenging to get the rods parallel into the domino connectors. A combination of rod bending and handling with a rod gripper helps achieve the contour necessary to set up the construct-to-construct configuration. After initial distraction is performed on the concavity of the scoliosis (Figure 3), the remainder of the spine should be exposed in a subperiosteal manner. Posterior column osteotomy releases will gradually enhance deformity correction by removing the posterior spinal elements, so long as the anterior columns of the corresponding motion segments are not fused together. Small and repetitive distraction maneuvers are applied over the course of the surgery until appropriate correction is achieved and final fusion may be performed. Upgoing rib hooks sometimes provide an excellent option for internal distraction (Figure 4 and Figure 5). If adequate correction is not achieved or there is concern about risk of neurological injury due to intraoperative neuromonitoring (IONM) changes, then a staged procedure may be performed at a later time.

### 3.3. Traditional Growing Rods

**Figure 6:**
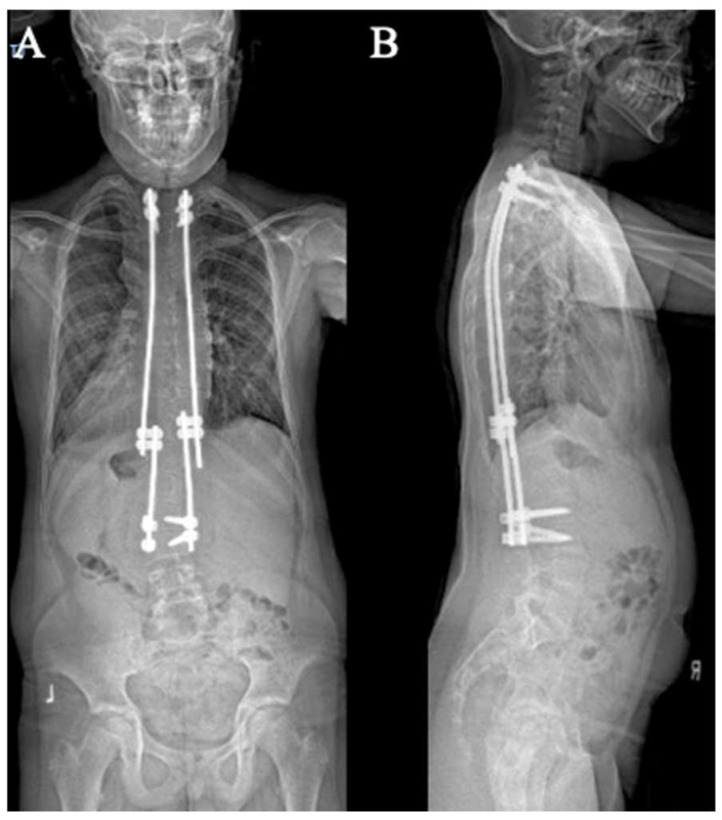
AP (**A**) and lateral (**B**) X-rays demonstrating the construct-to-construct internal distraction technique in a traditional growing rod construct.

The construct-to-construct internal distraction technique is the most common configuration for traditional growing rod constructs (Figure 6). The distal anchor points comprise two-level pedicle screw fixation. The last spinal level will represent the lower instrumented vertebra of the final fusion construct. The proximal anchor points may be either upgoing rib hooks or pedicle screws. We prefer pedicle screw fixation at upper instrumented vertebra, upper instrumented vertebra-1, and occasionally upper instrumented vertebra-2. It is important to keep in mind that these proximal pedicle screws will experience significant distraction forces with serial lengthening that could loosen the screws at the time of the final spinal fusion. A long titanium rod is passed under the muscle layer to engage into the proximal anchor points. The distal segment of this rod is connected to a lateral domino connector with significant overlap to allow for serial distraction in the future. A shorter rod is tunneled through the domino connector and engaged to the distal anchor points. A rod gripper is used to engage the longer, proximal rod approximately 1 cm above the domino connector (Figure 3). The set cap on the domino connector is loosened, and a distractor is placed between this connector and the rod gripper. Gradual distraction is applied in a controlled, “click-by-click” manner before the set cap is tightened to hold the correction.

### 3.4. Pedicle Subtraction Osteotomy

**Figure 7:**
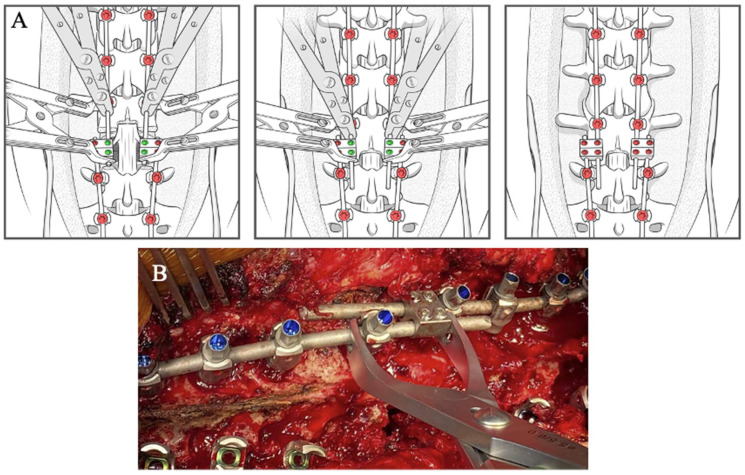
Step-by-step schematic of the construct-to-construct internal compression technique for closure of PSO. The green caps represent loose set caps. Red caps represent tight set caps (**A**). Intraoperative image adapted from Bourghli et al. showing a construct-to-construct internal compression technique to close a PSO site (**B**).

Pedicle subtraction osteotomies (PSOs) represent Schwab 3 and 4 osteotomies and are powerful techniques for correcting both sagittal and coronal fixed, spinal deformities [7]. The PSO is the first three-column osteotomy described in this technique paper. The construct-to-construct internal compression technique represents one method of closing this osteotomy. We recommend having several anchor points above and below the PSO level (three levels of fixation are preferred if possible). In the case of lumbar PSOs, a contoured rod is fixed to the distal anchor points and cantilevered over the osteotomy to connect into one or two domino connector(s) to form the construct-to-construct configuration [8]. A rod gripper attaches to this distal contoured rod, and a compressor is placed between the domino connector(s) and the rod gripper (Figure 7). Gradual compression is applied in a controlled, “click-by-click” manner before the set caps are tightened to hold the correction. This process is repeated on the contralateral side to finish closing down the osteotomy.

### 3.5. Vertebral Column Resection

Vertebral column resections (VCRs) represent Schwab 5 and 6 osteotomies in which one or more vertebral segments are completely removed, including the posterior elements, vertebral body, pedicles, lamina, and adjacent discs above and below the vertebral body [7]. The VCR is the second three-column osteotomy described in this technique paper. This procedure is performed to achieve maximal correction of rigid spinal deformities when other osteotomies are insufficient. VCR is typically reserved only for severe, rigid, and complex spinal deformities, such as congenital kyphosis or scoliosis with vertebral anomalies, severe angular deformities, or revision cases with persistent deformity. Corrections ranging up to 50–70% can be achieved in severe curves with the VCR technique [9]. The construct-to-construct internal compression technique is very useful to correct VCRs along the convexity of a severe scoliosis curve or on both sides of the spine in cases with a severe angular kyphosis. Alternatively, the construct-to-construct internal distraction technique can be useful to correct VCRs along the concavity of a severe scoliosis curve. Having several anchor points above and below the VCR level (three levels of fixation are preferred if possible) is critical to successfully and safely reduce the deformity. In VCR procedures involving a thoracolumbar fusion mass with weak screw fixation, internal compression and distraction techniques provide safer correction, avoiding the high risk of screw plowing that accompanies direct compression or distraction forces on individual screws.

### 3.6. Discussion

This report describes the utility and technical nuances of the construct-to-construct technique, a surgical adjunct that utilizes two rods joined by lateral domino connectors to enact powerful internal distraction or compression forces on the spine for achieving spinal deformity correction in severe scoliosis without requiring HGT or three-column osteotomies. The most common clinical application of this technique is for facilitating gradual correction of a severe and flexible scoliosis curve. Internal distraction using the construct-to-construct configuration is performed early in the surgery to take advantage of the viscoelastic properties of the spine as gradually increasing distraction forces are applied. This technique is also ubiquitously used in early-onset scoliosis and serves as the basis for internal distraction using traditional growing rods. Finally, the construct-to-construct technique can be used to compress or distract against coronal and sagittal plane spinal deformities requiring three-column osteotomies by generating powerful forces that are spread across multiple anchor sites.

Temporary internal distraction is a surgical technique used to correct severe scoliosis deformities that was first described by Buchoski et al. in 2006 [3,4]. Sixteen years later, the same group published a two-year minimum follow-up study, in which they reported outcomes of 51 patients with a mean age of 14.3 ± 3.5 years and mean follow-up of 5.8 ± 3.0 years [10]. The mean Cobb angle was 103° preoperatively and 20° at final follow-up, for a mean Cobb reduction of 81%. Eleven patients were treated in a staged fashion using temporary rods, and forty were treated in a single-stage procedure. The time between surgeries for staged internal distraction ranged from 11 to 31 days. During stage 1 of staged procedures, IONM alerts occurred in three (27.3%) cases. These changes were transient and did not correlate with any neurological deficits. During definitive fusion procedures, IONM alerts occurred in 10 (19.6%) cases. All of these cases except one (2%) returned to baseline after decreasing distraction, and no patient developed a neurological deficit. However, one neuromuscular patient developed delayed lower extremity weakness > 12 h after definitive fusion in spite of stable IONM. This deficit resolved after reoperation to decrease correction. This study underscores the powerful correction potential of internal distraction techniques and highlights its safety, as demonstrated by its low risk of neurological injury. If IONM changes did occur during internal distraction, most times, these changes were reversible with releasing distraction.

Less-invasive temporary internal distraction techniques (LI-TID) have also been utilized in the treatment of severe scoliosis to avoid preoperative HGT or vertebral column resections. This technique involves placing upper thoracic and lumbar pedicle screws in a minimally invasive fashion, similar to a growing-rod procedure. Posterior column osteotomies are performed around the fixation points; then, subcutaneous rods are tunneled and interconnected so graduate distraction can be applied. In 2020, Grabala et al. reported the results of 22 adolescents with severe idiopathic scoliosis (major curve > 90 degrees) who underwent LI-TID followed by staged pedicle screw instrumentation, with a minimum of 2 years of follow-up [11]. This technique provided 51% correction of the major curves and improved the percentage of the predicted forced vital capacity by 49%. Of note, five (22.7%) patients experienced an IONM change without postoperative neurological deficits. A more recent study by Grabala in 2024 compared the outcomes of 62 pediatric patients with severe idiopathic scoliosis who underwent HGT (n = 20) and LI-TID (n = 42) before final fusion [12]. Aside from improvements in radiographic Cobb angle corrections in the coronal and sagittal planes, the authors demonstrated a 76% reduction in rib hump deformity, improvement in predicted forced expiratory volume in 1 s by 25–56%, and improvement in forced vital capacity by 35–65% in the LI-TID cohort. This cohort also reported an improvement in quality of life, as measured by the SRS-22r scale, further emphasizing the safety and effectiveness of internal distraction techniques for correcting severe scoliosis curves.

An important historical lesson reported by our predecessors that cannot be forgotten is the iatrogenic and preventable cause of paralysis when excessive traction is exerted on the spinal cord [13]. Neuromonitoring changes during pediatric spinal deformity surgery range from 9.1 to 27.8% [14,15]. For these important reasons, experts who utilize TID for spinal deformity surgery consider reliable neuromonitoring to be a vital sign similar to oxygen saturation, heart rate, and blood pressure [16]. A change in neuromonitoring after internal distraction maneuvers can help provide an early warning of a potential neurologic injury and should prompt the immediate attention of the surgical team. In 2014, Skaggs et al. showed that neuromonitoring changes are common and reversible with TID for pediatric patients with severe scoliosis [16]. This study evaluated 22 patients with a mean age of 14 years who underwent TID and achieved a mean correction of 62 degrees. Nine of these patients had staged procedures separated by a mean of 7 days. Of these 22 patients, 9 (41%) had IONM changes. These patients with IONM achieved the same amount of mean final correction as did patients who did not have IONM alerts. There were no postoperative clinical neurological deficits in any of the nine patients. Distraction forces were released after IONM signal loss, and this immediately reversed the drop in transcranial motor evoked potentials (tc-MEPs) in each patient. One patient had a persistent drop in tc-MEPs in the left leg after the third attempt of gradually decreasing distraction during their first surgery. The temporary rod was removed without improvement in tc-MEPs, but the patient awoke without a neurological deficit. The second surgery proceeded uneventfully without any IONM changes. The authors contend that a lack of reliable IONM data represents a contraindication to use of TID techniques. They recommend the following protocol after a loss of IONM signals that the surgeon feels to be a true reflection of a potential neurological injury: (1) release of distractive forces on the temporary rod with shortening to wherever the rods reside without further manipulation; (2) elevation of the MAP to 80 mm Hg or more; (3) cessation of distraction that day, with the connectors locking the length of the rod in place; (4) consideration of return to surgery at least 1 week later for the final fusion [16].

Another potential complication of construct-to-construct internal distraction, in addition to triggering IONM changes, is pedicle plowing and loosening at the proximal and distal anchor points. This risk of pedicle screw plowing during internal distraction is why we favor having at least two (or more) anchor points at the proximal and distal ends to set up this distraction technique. Having multiple proximal and distal anchor points helps prevent plowing of pedicle screws by distributing the distractive and compressive forces across several motion segments along the spine. To further hedge against the risk of pedicle screw plowing, we recommend against utilizing either upper or lower instrumented vertebra as anchor points in the construct-to-construct configuration. This setup avoids losing critical fixation points at the ends of the spinal construct if screw plowing does occur. This potential complication of screw plowing and loosening can be further minimized by ensuring accurate pedicle screw placement, resulting in strong fixation and by performing serial, careful distraction forces in a “click-by-click” manner. We believe the benefits of construct-to-construct distraction outweigh its potential complication risks, so long as the technique is executed in a thoughtful and judicious manner. The principal advantage of this technique is improving deformity correction in patients with severe, flexible scoliosis curves without requiring HGT or three-column osteotomy.

## 4. Conclusions

Construct-to-construct internal distraction and compression techniques are powerful methods to correct severe scoliosis curves, serially distract traditional growing rod constructs, and close three-column osteotomies.

## Data Availability

Data sharing not applicable to this article as no datasets were generated or analyzed during the current study.
